# Short‐term Clinical Outcome of Dual Plate Fixation in the Treatment of Proximal Humerus Fractures with Calcar Comminution

**DOI:** 10.1111/os.13601

**Published:** 2022-12-08

**Authors:** Junyang Liu, Peng Cui, Xiaoming Wu, Lei Han, Guangyu Wang, Jingming Dong

**Affiliations:** ^1^ Department of No. 2 Upper Extremity Traumatology Tianjin Hospital Tianjin China; ^2^ Department of Orthopaedic Traumatology, Trauma Center, Shanghai General Hospital Shanghai Jiao Tong University School of Medicine Shanghai China

**Keywords:** Calcar, Dual Plate, Fracture Fixation, Shoulder Fracture

## Abstract

**Objective:**

Calcar comminution has been considered to be the main cause of the failure of internal fixation and fracture nonunion in proximal humerus surgery. Anatomical reduction and increasing the strength of internal fixation is the key to success. The purpose of this study was to investigate the short‐term clinical effect of dual plate fixation in the treatment of proximal humeral fractures with calcar comminution.

**Methods:**

The data of 37 patients with proximal humeral fractures with calcar comminution, treated in our departments from July 2018 to April 2020, were retrospectively analyzed. These patients were treated with anterior plate and lateral PHILOS plate, and followed up for more than 12 months, including 25 cases in Tianjin Hospital and 12 cases in Shanghai General Hospital. The patients included 12 males and 25 females, their age was 54.89 ± 13.59 years (range from 32–79 years), and 21 patients had dominant hand injury. According to the Neer classification, there were 11 two‐part fractures, 22 three‐part fractures, and four four‐part fractures. The range of motion of the shoulder joint, visual analog scale (VAS), American Shoulder and Elbow Surgeons Shoulder Score (ASES), Constant–Murley shoulder score, neck‐shaft angle, anterior–posterior angle, and other complication scores were recorded at the last follow‐up.

**Results:**

All 37 patients were followed up after operation, and the follow‐up time was 21.81 ± 7.35 months (range from 12–36 months). The fractures of all 37 patients had healed at the last follow‐up visit. The neck‐shaft angle measured immediately after operation was 132.59° ± 8.34°, and the neck‐shaft angle measured at the last follow‐up visit was 132.38 ± 8.53°. The anterior–posterior angle measured immediately after surgery was 3.45° ± 0.81°, and the anterior–posterior angle at the last follow‐up visit was 3.66° ± 0.77°. The range of motion of the shoulder joint was as follows: the shoulder joint could be forward elevated by 158.11° ± 13.09° (range: 140°–180°), rotated externally by 38.38° ± 7.55° (range: 20°–45°), and internally rotated to T4‐L4 level. The VAS score was 0.46 ± 0.87 (range: 0–3), the ASES was 86.58 ± 8.79 (range: 56.7–100), and the Constant–Murley score was 88.76 ± 8.25 (range: 60–100). Thirty‐three cases were excellent, and four cases were good. No obvious complications occurred.

**Conclusion:**

The combination of anterior plate and lateral PHILOS plate in the treatment of proximal humeral fractures with calcar comminution can achieve stable fixation, and the postoperative clinical and imaging outcome was satisfactory. Firstly, the anterior plate can provide temporary stability when the Kirschner wires are removed, which can provide space for lateral plate placement during fracture reduction and fixation. Secondly, additional support by the anterior plate can provide higher stability in complex fractures with calcar comminution.

## Graphical abstract

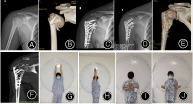

(A) and (B) are the frontal X‐ray and computed tomography (CT) images showing right proximal humerus fracture with comminuted calcar. (C) X‐ray image taken immediately after the operation, showing that the proximal humerus is well‐reduced and the neck‐shaft angle is restored with dual plates. (D–F) X‐ray images in the anteroposterior (AP) view and the CT image showed plate positions were satisfactory and bone union was successful. (G–J) Shows that 1 year after the operation, the functional recovery was satisfactory.

## Introduction

Proximal humeral fracture is a common clinical fracture, which accounts for 5%–6% of all fractures, and is the third most common fracture in the elderly. About 20% of proximal humeral fractures require surgical treatment.^1^, ^2^, ^3^ Currently, the surgical treatment for proximal humeral fractures mainly involves open reduction and internal fixation and shoulder replacement. Shoulder replacement should be considered when the comminuted fracture is difficult to reduce, the rotator cuff cannot be repaired, or the blood supply to the humeral head is found to be severely compromised during preoperative assessment.^4^ In the vast majority of the remaining cases, locking plates are widely used and have become the gold standard due to their anatomical shape, low profile, suture holes, and angular stabilization.^5^ However, despite these advantages, the locking plate technique has been reported to have a high complication rate (9.9%–35%) in the surgical management of proximal humeral fractures.^6^, ^7^, ^8^, ^9^ The lack of anatomical reduction and support of the medial calcar has been considered to be the main cause of increased failure rate and nonunion.^9^, ^10^, ^11^

The common methods used to strengthen the calcar support include locking additional calcar screws, fibula allograft, and dual plate fixation. Calcar screws with locking plates can increase medial column support, but this outcome has a low possibility in short‐stature women. Besides, its mechanical stability is inferior to fibula allograft or dual plate fixation.^12^ In reported studies, fibula allograft has achieved good results, maintained stability and reduced the incidence of complications.^13^, ^14^, ^15^ However, a revision procedure is challenging when absorption of large tubercle or necrosis of the humeral head has occurred. Only a few reports on the dual plate technique have been published in the literature, and the placement of the additional plate varies.

The purpose of this study is to show the outcome of using the anterior locking plate technique to assist the lateral anatomical locking plate technique in the treatment of proximal humeral fractures with calcar comminution since 2017. This study conducted a retrospective analysis of this set of data with the following objectives: (1) To evaluate the efficacy of the dual plate technique in the treatment of proximal humeral fractures from the clinical and imaging aspects; (2) To discuss the surgical techniques and advantages of dual plate fixation.

## Materials and Methods

### Inclusion and Exclusion Criteria

Inclusion criteria were as follows: (1) proximal humerus fracture with calcar comminution (calcar is splintered or crushed); (2) treated with anterior locking plate assisted with a lateral anatomical locking plate; (3) the main evaluation indicators were neck‐shaft angle, pain, and shoulder joint function; (4) patient's age from 18 to 80 years; (5) at least 12 months of follow‐up. Exclusion criteria as follows: (1) pathological fracture of proximal humerus; (2) preoperative combined nerve and vascular injury.

### General Information

A total of 240 cases of proximal humerus fractures were surgically treated in our department from July 2018 to April 2020. Based on the inclusion and exclusion criteria, 37 cases were included in this study, of which 12 were male and 25 female, with an average age of 54.89 ± 13.59 years (ranging from 32–79 years). The injury mechanisms included 22 pedestrian accidents, 13 traffic accidents, and two high fall accidents. All cases were closed fresh injuries; 21 were in the dominant side; 11 had concomitant injuries, including six cases with shoulder dislocation, all of which were treated in emergency by reduction. According to the Neer classification, 11 cases (29.7%, 11/37) were two‐part fractures (humerus surgical neck fracture); 22 (59.5%, 22/37) had three‐part fractures (humerus surgical neck and greater tuberosity fracture); and four (10.8%, 4/37) had four‐part fractures (humerus surgical neck, greater and lesser tuberosity fracture). This study was approved by Tianjin hospital review board (2020–0035), and patients gave their informed consent.

### Surgical Method

#### Position and Approach

After general anesthesia, the fluoroscopic operating table was used to bring the torso up to a beach chair position while the head was secured to an appropriate headrest position with a head tilt of 30°, and a pillow was placed under the shoulders to facilitate exposure of the shoulder joints. The deltoid pectoralis approach was used and the cephalic vein was exposed and protected. Attention was paid to ligating the lateral branch of the cephalic vein and pulling it to the medial side. After separating the deltoid muscle and the pectoralis major muscle, the clavicular fascia was opened, the long head of the biceps tendon was found, the intertubercular groove was identified accordingly, and the locations of the greater tuberosity and lesser tuberosity were confirmed. Nonabsorbable sutures (5#Ethibond) were used to fix the tendon‐bone junction at the greater and lesser tuberosity separately. The rotator interval was released. The long head of the biceps was released from its supraglenoid tubercle insertion. Then, the medial border of the intertubercular sulcus was exposed by stripping off less than 5 mm of the insertion of the pectoralis major. The blood clots and embedded soft tissue were cleaned, taking care to protect the rotator cuff tissue.

#### Fracture Reduction and Temporary Fixation

First, the greater (lesser) tuberosity and humeral head were repositioned using nonabsorbable sutures and point reducer, and multiple 1.6‐mm Kirschner wires were temporarily fixed under fluoroscopic guidance. Then, the proximal humerus and the humeral shaft were reduced. Due to the comminuted calcar region and the lack of medial support, attention was paid to avoid the varus deformity.

#### Placement of Anterior Plate

The 2.7‐mm micro‐locking plate (Synthes Inc., Paoli, PA, USA) was placed under the medial lesser tuberosity to function as a Buttress plate. After confirming the position by fluoroscopy, two locking screws were fixed at the distal and proximal ends.

#### Placement of the Lateral Plate

A PHILOS locking anatomical plate (Synthes Inc.) of the proximal humerus was used for the lateral plate. A nonabsorbable suture was passed through the suture hole in the plate. It is important to note that the position of the plate should be located 5–8 mm below the top of the greater tuberosity and 2–4 mm lateral to the intertubercular groove. A countersunk screw and E countersunk screw were then fixed to the proximal part, and a locking screw was fixed into the distal part of the fracture through the plate hole. After confirming the position and reduction effect by fluoroscopy again, 4–5 locking screws were inserted again, and two locking screws were inserted at the distal end. Both the greater tuberosity (GT) and lesser tuberosity (LT) were fixed with the preset non‐absorbable suture. The distal part of the long head of the biceps tendon was sutured to the short head of the biceps. Ultimately, the wound was irrigated, sutured layer by layer, and closed.

### Postoperative Treatment and Rehabilitation

The shoulder brace (90° bent elbow with the forearm in neutral position) was routinely worn for protection by all patients. On the first postoperative day, patients were assisted at the bedside with active movements of the elbow, wrist, and fingers, and initial pendulum‐like movements of the shoulder joint. After the operation, the passive functional exercise of the shoulder joint was performed under the guidance of the physical therapist doctor within 6 weeks postoperatively. A follow up with X‐ray exam took place at 6 weeks postoperatively. After the fracture site was stable and the callus grew, active functional exercise was started. Strength training was gradually increased after 3 months.

### Evaluation Indicators

#### Imaging Measurement

1. Measurement of the neck‐shaft angle: On the frontal X‐ray image of the shoulder joint, the angle between the vertical line of the anatomical neck of the humeral head and the long axis of the humeral shaft was the neck‐shaft angle. A neck‐stem angle of 120–150° was defined as anatomical reduction, 110–120° was defined as mild varus deformity, less than 110° was defined as severe varus deformity, and greater than 150° was defined as valgus deformity.^16^
2. Anteroposterior angulation: Lateral radiographs of the humerus was used to evaluate the anteroposterior angulation of the proximal humerus. An anteroposterior angulation of less than 5° degrees was excellent, 5–10° was good, and greater than 10° was poor.
3. Internal fixation failure: the internal fixation was considered a failure when the change of the neck‐shaft angle in the frontal X‐ray image (immediately postoperatively) and the neck‐shaft angle at the last follow‐up visit was more than 5°, or the change of the anteroposterior angulation was greater than 5° in the lateral X‐ray image. The displacement of the plate and the bone or the fracture of the plate were also checked.
4. Fracture healing: When the X‐ray image showed that the fracture line was blurred or disappeared, and there was a continuous callus passing through the fracture line, it was considered as a healed fracture.
5. Necrosis of the humeral head: At the last follow‐up visit, X‐ray images of the anterior, posterior, and axillary positions of the shoulder joint were used for evaluation. According to the Cruess classification system, the X‐ray images were evaluated to determine whether there were sclerosis or decreased bone density, crescent shape, trabecular bone destruction, subchondral bone collapse, and secondary joint degeneration.^17^

#### Visual Analog Scale (VAS)

Pain was assessed by the VAS score. This scale is commonly graded from 0 to 10, where 0 is completely painless, 1–3 is mild pain, 4–6 is moderate pain, and 7–10 is severe pain.

#### Range of Motion of the Shoulder Joint

The angle of flexion and lifting the shoulder joint, the angle of the lateral external rotation, and the height at which the thumb touches the back during internal rotation were recorded, and shoulder mobility was assessed.

#### The American Shoulder and Elbow Surgeons Shoulder Score (ASES)

The ASES included: pain (50%) and life function (50%). The full score was 100 points, and the higher the score, the better the function of the shoulder joint.

#### The Constant–Murley Score (CMS)

The function of the shoulder joint was assessed using the CMS. For evaluation, the score was divided into pain (15 points), daily activities (20 points), active range of motion (40 points), and muscle strength (25 points). The total score was 100 points, and the higher the score, the better the function. The overall satisfaction was judged according to the CMS: higher than 75 was considered excellent, 50–75 was considered good, and lower than 50 was considered poor.

### Statistical Analysis

Statistical analysis was performed using the SPSS 21.0 statistical software (IBM Corporation, Armonk, NY, USA). The measurement data (VAS score, flexion and lift angle, external rotation angle, ASES, CMS, and neck‐shaft angle) conforming to normal distribution were expressed as the mean ± standard deviation. All data were analyzed using ANOVA, repeated measures ANOVA and a p‐value less than 0.05 was considered statistically significant.

## Results

### Intraoperative Situation

The 37 cases in this study were treated by one group of surgeons. The average operation time was 127.5 min (range: 75–185 min). The intraoperative blood loss was 180.0 mL (range: 100–350 mL), without blood transfusion. Intraoperative exploration found no complete anterior humeral circumflex artery below the lesser tuberosity.

### Clinical Outcomes

All 37 patients were followed up for 21.81 ± 7.35 months (range: 12–36 months).

#### Imaging Results

**Fig. 1:**
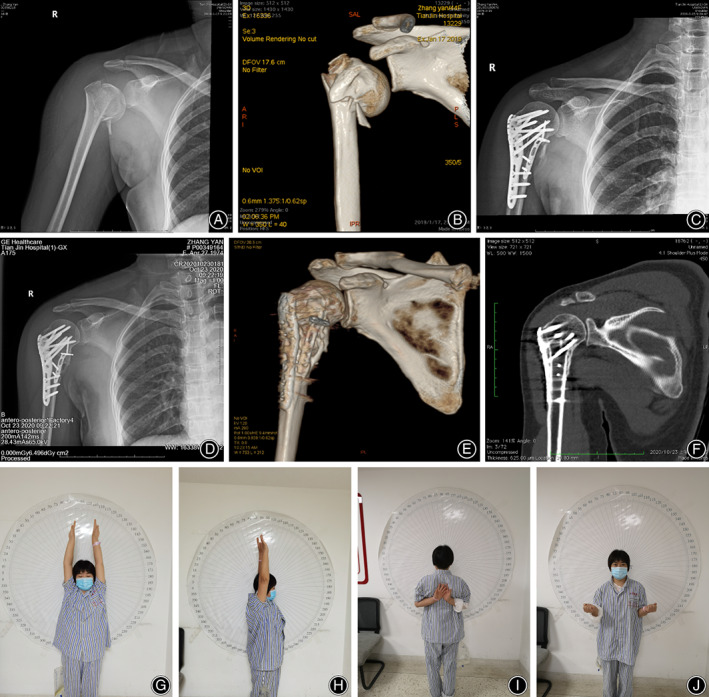
The patient was a 44‐year‐old female with a proximal humerus fracture (Neer classification: three‐part fracture). (A, B) Frontal X‐ray images and computed tomography (CT) images of the right shoulder joint after a fall on the right shoulder, showing a comminuted fracture of the proximal end of the right humerus and a comminuted calcar. (C) X‐ray image taken immediately after the operation, showing that the proximal humerus is well‐reduced and the neck‐shaft angle is restored. (D–F) X‐ray images in the anteroposterior (AP) view and the CT image showed: plate position was satisfactory and bone union was successful. (G–J) Show that 1 year after the operation, the shoulder joint could be flexed and lifted up to 180°, the internal rotation reached the T4 level, the lateral external rotation was 30°, and the functional recovery was satisfactory.

All fractures healed in an average of 4.85 months (range: 3–6 months). There was no absorption of greater tuberosity or lesser tuberosity, delayed union or nonunion, and no humeral head necrosis occurred. The neck‐shaft angle of the 37 patients immediately after operation was 132.59° ± 8.34°, and at the last follow‐up visit was 132.38° ± 8.53°. There was no significant difference between the two time points (immediately after operation and last follow‐up visit). The anteroposterior angulation of the 37 patients: the anteroposterior angulation was 3.45° ± 0.81° immediately after operation, and 3.66° ± 0.77° at the last follow‐up visit. There was no significant difference between the two time points (*p* = 0.195). None of the patients had internal fixation failure (Figure 1).

#### Functional Score

The VAS scores of the 37 patients decreased gradually over time, and the pain was relieved. In particular, it was 3.62 ± 1.01 (range: 2–5 points) at 3 months after operation, 1.78 ± 0.89 (range: 0–4 points) at 6 months after operation, and the last VAS score was 0.46 ± 0.87 points (range: 0–3 points). The differences were statistically significant between 3 months and 6 months after operation and between 6 months after operation and the final follow‐up visit (*p* all below than 0.05, Table 1). At the last follow‐up visit, the Neer VAS score was 0.73 ± 1.01 points for two‐part fractures, 0.23 ± 0.69 points for three‐part fractures, and 1.01 ± 1.16 points for four‐part fractures. The difference among the three was not statistically significant (*p* = 0.649, Table 2). At the last follow‐up visit, 28 patients had no pain, and nine patients had pain after heavy activity, which was relieved by rest. None of the patients had sleep disturbance or nocturnal pain.

**TABLE 1:** Comparison of CMS, ASES, and VAS scores of the 37 patients at 3 and 6 months after operation and at last follow‐up visit (points)

Time	Cases	CMS	ASES	VAS score
3 months after surgery	37	57.95 ± 8.77	54.02 ± 9.08	3.62 ± 1.01
6 months after surgery	37	74.76 ± 9.36	72.30 ± 8.28	1.78 ± 0.89
Last follow‐up	37	88.76 ± 8.25	86.58 ± 8.79	0.46 ± 0.87
*F* value		29.55	23.60	19.34
*p* value		<0.05	<0.05	<0.05

**TABLE 2:** Comparison of CMS, ASES, and VAS scores of the Neer's two‐part, three‐part, and four‐part fractures at the last follow‐up visit (points)

Groups	Cases	CMS	ASES	VAS score
Two‐part fracture	11	88.91 ± 8.73	86.64 ± 9.57	0.73 ± 1.01
Three‐part fracture	22	87.11 ± 7.98	87.11 ± 7.98	0.23 ± 0.69
Four‐part fracture	4	86.75 ± 11.59	86.75 ± 11.59	1.01 ± 1.16
*F* value		0.127	0.270	2.223
*p* value		0.881	0.765	0.124

After operation, the ASES gradually increased with time. The average ASES was 54.02 ± 9.08 points (range: 37–70 points) at 3 months after the operation, 72.30 ± 8.28 points (range: 51–85 points) at 6 months after the operation, and 86.58 ± 8.79 points (range: 57.7–100 points) at the last follow‐up visit. There were statistically significant differences in the ASES both between 3 and 6 months postoperatively and between 6 months and the last follow‐up visit (all *p* < 0.05, Table 1). At the last follow‐up visit, the ASES was 86.64 ± 9.57 points for two‐part fractures, 87.11 ± 7.98 for three‐part fractures, and 83.53 ± 12.71 for four‐part fractures. The difference among the three groups was not statistically significant (*p* = 0.512, Table 2).

The CMS gradually increased with time. It was 57.95 ± 8.77 points (range: 40–70 points) at 3 months after the operation, 74.76 ± 9.36 points (range: 50–90 points) at 6 months after the operation, and 88.76 ± 8.25 points (range: 60–100 points) at the last follow‐up visit. There were statistically significant differences in the CMS between 3 months and 6 months postoperatively, and between 6 months and the last follow‐up visit (all *p* < 0.05, Table 1). At the last follow‐up visit, Neer's CMS was 88.91 ± 8.73 for two‐part fractures, 87.11 ± 7.98 for three‐part fractures, and 86.75 ± 11.59 for four‐part fractures. The difference among the three groups was not statistically significant (*p* = 0.439, Table 2).

At the last follow‐up visit, the forward flexion was 161.20° ± 13.01° (range: 140°–180°), the external rotation was 37.60° ± 7.65° (range: 20°–45°), the internal rotation was to T4‐L4 level, and 33 patients were rated as excellent, while the remaining four patients were rated as good.

#### Complications

There was no neurovascular injury, no incision infection, no loosening and fracture of internal fixation, no screw penetration of the humeral head, and no necrosis of the humeral head.

## Discussion

Our results showed that dual plate fixation significantly assisted the reduction of the fracture and enhanced the structure of the internal fixation. Patients were able to achieve satisfactory treatment outcomes.

### Advantages of the Dual Plate Fixation of the Proximal Humerus

In this study, 37 cases of proximal humeral fractures with calcar comminution were treated with anterior plate and lateral PHILOS plate. The results confirmed, according to the postoperative neck‐shaft angle, anteroposterior angulation without obvious changes, fracture healing and functional score, that the dual plate fixation provided good stability, and the patients achieved satisfactory function.

Proximal humeral fractures with calcar comminution are difficult to treat surgically, and are more likely to lead to failure of the internal fixation. The comminution of the calcar can be used as an independent predictor of the success of the locking plate fixation.^11^ Russo *et al*. proposed that the calcar should be defined as the fifth most important part besides the greater and lesser tuberosities, the humeral head, and the diaphysis. In addition, the surgical plan should be formulated according to the fracture of the calcar.^18^ The fixation or reconstruction of the calcar is very important for the fixation of comminuted fractures.^10^ In clinical practice, the commonly used techniques for strengthening the medial column include talar screws, fibula bone grafting, and dual plate fixation techniques. The placement of calcar screws cannot be guaranteed. For example, in this study, we were unable to place calcar screws in five patients, and four of them were elderly women with small stature. Lee *et al*. reported their results of 45 unstable proximal humeral fractures treated with locking plates. The mean head‐shaft angle was 132.4° after the operation and decreased to 127.7° at final follow‐up. Five patients showed considerable loss of fixation.^19^ In our case series, the neck‐shaft angle of the 37 patients immediately after operation was 132.59°, and at the last follow‐up visit was 132.38. There was no patient considered as loss of fixation. Augmentation with an endosteal fibular allograft improves construct stability. According to a new review, the use of FA obtained better outcomes in the clinical scales in all comparative studies, improved their range of motion by an average of 21° for flexion and 19° for abduction. FA prevented varus collapse of the fracture, decreasing changes in the NSA throughout the follow‐up. But 13 cases of AVN (3%) were present in the FA group. Patients who developed AVN either refused treatment or followed conservative treatment.^20^ When the humeral head is necrotic, if the orthopaedic surgeon wants to do a shoulder replacement, reaming of the humerus canal in the presence of an existing intramedullary fibular graft is challenging because the fibula is a dense cortical bone and is typically well‐integrated. Lu *et al*. reported good results in the treatment of obsolete proximal humeral fractures with double plates.^21^, ^22^ Compared to those other techniques, the advantage of dual plate fixation is obvious for the following reasons.First, during the reduction process, the anterior plate can provide stability by fixing the lesser tuberosity, humeral head, and diaphysis, which allowed the Kirschner wire temporary fixation to be removed and provided space for lateral plate placement.Second, the anterior plate provides additional biomechanical strength for fixation. In a biomechanical study of proximal humeral fractures, He *et al*. compared multiple methods of increasing the strength of the internal fixation and found that dual plates provided greater support than lateral plates alone.^12^ In addition, Park *et al*. suggested that the addition of internal fixation plates could prevent necrosis of the humeral head by providing solid internal fixation.^23^ In this study, there was neither delayed union nor nonunion, suggesting that dual plates provide good biomechanical strength for fixation.

Third, in this study, no complete anterior humeral circumflex artery was found under the lesser tuberosity in the 37 patients, and the microplate fixation below the small tuberosity can prevent excessive medial placement from further damaging the blood supply of the comminuted bone fragments of the medial calcar and the posterior circumflex humeral artery. As a result, since the posterior circumflex humeral artery is considered to be the main source of blood supply to the humeral head,^24^ none of the patients had humeral head necrosis at the last follow‐up visit.

### Differences from Other Dual Plate Techniques

First, compared with the medial plate, the anterior plate is easier to place, and there is no need to strip too much soft tissue, which is especially important for the medial calcar, itself a comminuted fracture, in which the excessive separation would cause the comminuted bone to lose blood supply. When using a medial buttress plate for calcar comminution, it is possible to damage more soft tissue, which leads to potential instability and impingement. In addition, the medial approach may damage the musculocutaneous and axillary nerves.

Second, compared with the posterior plate, the anterior plate plays an important role in the fixation after reduction, which can help stabilize the fracture end and play a temporary fixation role. The posterior plate needs to be added again after the lateral plate is fixed, which is less important. In addition, there is the possibility of damaging the posterior circumflex humeral artery and affecting the external rotation.

Third, compared with the plate in the anterior bicipital groove, the plate placed under the lesser tuberosity is closer to the humeral head, with better bone mass and easier fixation of the humeral head. In addition, the bicipital groove is often fractured or close to the fracture line and cannot be effectively fixed.

### Strengths and Limitations

This study has the following strengths: The surgery for proximal humeral fractures with calcar comminution has failed during fracture reduction and fixation. The key to the surgery is to remove the Kirschner wire and place a lateral plate. This study firstly proposed using of anterior plate to stabilize this process. At the same time, the anterior plate can increase the final stability.

This study has the following limitations: (1) This is a retrospective study, and a prospective clinical study should be designed for the follow‐up; (2) There is no control group in this study, and further research should compare the efficacy of fibula bone grafting and dual plate fixation treatment; (3) This study is a single‐center study with a small sample size. Therefore, to confirm the advantages of dual plates, a multi‐center study with a large sample is required.

### Conclusion

In conclusion, restoring the support of the medial column is critical for proximal humeral fractures with calcar comminution. The use of the anterior microplate to assist the lateral anatomical locking plate can provide sufficient stability during and after the operation, which allows patients to perform early functional exercise, to achieve a satisfactory curative effect.

## Conflicts of Interest

The authors declare that they have no conflict of interest related to the publication of this manuscript. All authors agree with the manuscript. Funding Information: No funding was received for this research.

## Author's Contribution

Junyang Liu acquired and wrote the manuscript, Peng Cui analyzed data, Xiaoming Wu and Jingming Dong performed the surgery. Lei Han and Guangyu Wang collected data. We sincerely thank all the patients and medical stuff in this study for their support.

## Ethics Statement

This study was approved by Tianjin Hospital review board (2020–0035).
